# Dose-response relationship of *Ralstonia solanacearum* and potato in greenhouse and *in vitro* experiments

**DOI:** 10.3389/fpls.2022.1074192

**Published:** 2022-12-20

**Authors:** Carina Eisfeld, Jack F. Schijven, Pieter Kastelein, Boris M. van Breukelen, Gertjan Medema, Jouke Velstra, Peter F. M. Teunis, Jan M. van der Wolf

**Affiliations:** ^1^ Department of Water Management, Faculty of Civil Engineering and Geosciences, Delft University of Technology, Delft, Netherlands; ^2^ Department of Statistics, Informatics and Modelling, National Institute of Public Health and the Environment, Bilthoven, Netherlands; ^3^ Department of Earth Sciences, Faculty of Geosciences, Utrecht University, Utrecht, Netherlands; ^4^ Department of Biointeractions and Plant Health, Wageningen Plant Research, Wageningen, Netherlands; ^5^ Water Quality & Health, KWR Water Research Institute, Nieuwegein, Netherlands; ^6^ Acacia Water B.V., Gouda, Netherlands; ^7^ Center for Global Safe Water, Sanitation and Health, Hubert Department of Global Health Rollins School of Public Health Emory University, Atlanta, GA, United States

**Keywords:** dose-response, irrigation water quality, infectivity, *Solanum tuberosum*, bacterial wilt, brown rot, risk assessement, *Ralstonia solanacearum*

## Abstract

*Ralstonia solanacearum* is the causative agent of bacterial wilt of potato and other vegetable crops. Contaminated irrigation water contributes to the dissemination of this pathogen but the exact concentration or biological threshold to cause an infection is unknown. In two greenhouse experiments, potted potato plants (*Solanum tuberosum*) were exposed to a single irrigation with 50 mL water (non-invasive soil-soak inoculation) containing no or 10^2^ – 10^8^ CFU/mL *R. solanacearum*. The disease response of two cultivars, Kondor and HB, were compared. Disease development was monitored over a three-month period after which stems, roots and tubers of asymptomatic plants were analyzed for latent infections. First wilting symptoms were observed 15 days post inoculation in a plant inoculated with 5x10^9^ CFU and a mean disease index was used to monitor disease development over time. An inoculum of 5x10^5^ CFU per pot (1.3x10^2^ CFU/g soil) was the minimum dose required to cause wilting symptoms, while one latent infection was detected at the lowest dose of 5x10^2^ CFU per pot (0.13 CFU/g). In a second set of experiments, stem-inoculated potato plants grown *in vitro* were used to investigate the dose-response relationship under optimal conditions for pathogen growth and disease development. Plants were inoculated with doses between 0.5 and 5x10^5^ CFU/plant which resulted in visible symptoms at all doses. The results led to a dose-response model describing the relationship between *R. solanacearum* exposure and probability of infection or illness of potato plants. Cultivar Kondor was more susceptible to brown-rot infections than HB in greenhouse experiments while there was no significant difference between the dose-response models of both cultivars in *in vitro* experiments. The ED_50_ for infection of cv Kondor was 1.1x10^7^ CFU. Results can be used in management strategies aimed to reduce or eliminate the risk of bacterial wilt infection when using treated water in irrigation.

## 1 Introduction

The *Ralstonia solanacearum* species complex causes bacterial wilt and comprises three different species, namely *R. solanacearum*, *R. pseudosolanacearum* and *R. syzigii* (Safni et al., 2014). Together they affect more than 200 plant species present in tropical to temperate climates. Hosts include *Solanaceous* crops, groundnut, banana and plantain, weeds, some tree species and ornamental plants (Hayward and Hartmann, 1994). In temperate climates, potato (*Solanum tuberosum*) production is affected by brown-rot, caused by *R. solanacearum* phylotype II (race 3 biovar 2) which is a strain adapted to cooler regions (Janse, 1996). As a result of a high number of potato brown rot outbreaks in Europe during the 1990’s, the pathogen complex had been put under quarantine status in the European Union with the aim to prevent its spread and eradicate the pathogen after outbreaks (Anonymous, 1998; Anonymous, 2006).

The emergence of brown rot has predominantly been associated with the use of (latently) infected seed tubers. Trading of seed and ware potatoes forms the highest risk of disease spread internationally (EFSA et al., 2019). For example, the first occurrence of brown rot in the Netherlands was detected in a ware potato field in 1992, presumably introduced through infected non-certified seed tubers (Janse et al., 1998). Natural dispersal of brown rot is mainly caused by contaminated irrigation water as surface water used in irrigation can become contaminated with run-off water from *R. solanacearum* infected agricultural fields or through contact with effluent water coming from potato processing industry (EFSA et al., 2019). *R. solanacearum* has been repeatedly detected in surface waters in the Netherlands and other European countries and disease outbreaks were directly linked to the use of contaminated surface water in irrigation (Janse, 1996; Wenneker et al., 1999). Moreover, *R. solanacearum* survives in plants such as bittersweet nightshade (*Solanum dulcamara*) and to a lesser extent in stinging nettle (*Urtica dioica*). They often grow along ditches next to agricultural fields from where the bacterium is released into the environment in high numbers (Olsson, 1976; Elphinstone et al., 1998). Eventually, this led to an irrigation ban on the use of surface water for (seed) potato production in the Netherlands and other European countries. Although this measure has effectively reduced the number of brown rot incidences (Janse, 2012) farmers were confronted with increased water scarcity. Rainfall can be insufficient to meet crop water requirements during the cropping season. In potato production, irrigation is essential to ensure a high tuber yield and potatoes are specifically sensitive to water stress during tuber initiation (Alva, 2008). Water scarcity in agriculture will further increase since surface water is not available for irrigation anymore and groundwater extraction is often restricted or prohibited as a result of climate change (Eyring et al., 2021). Consequently, farmers are looking for different fresh water sources which often include the recycling of irrigation water as done in greenhouse cultivation or the treatment of water with lower quality (e.g. treated wastewater) (Hong and Moorman, 2005). Additionally, more efficient irrigation systems like drip irrigation can be installed to reduce water use (Shock et al., 2002). Although these water management strategies help to overcome water shortages, (plant) pathogenic micro-organisms and other (agro-)pollutants may accumulate in water recycling systems (Ristvey et al., 2019). Therefore, treated water needs to meet quality requirements for irrigation water. Populations of pathogenic organisms need to be lower than the biological threshold which is the minimum inoculum level or dose which is required to cause a response (infection) in the host (Lamichhane and Bartoli, 2015).

In nature, a minimum pathogen concentration is required to establish an infection which had to withstand detrimental environmental conditions and the host defense system. Under optimal conditions for the pathogen, however, the concentration to infect a host may be as low as a single cell. Information on the biological threshold for plant pathogens in irrigation water is scarce despite its relevance (Stewart-Wade, 2011). For *R. solanacearum*, most infection studies analyzed the resistance of potato cultivars or the virulence of different *R. solanacearum* strains using high concentrations of about 10^6^-10^8^ CFU/mL [e.g. Lebeau et al. (2010); Aliye et al. (2015); Mori et al. (2015)]. Bowman and Sequeira (1982) tested wilt-resistant potato clones against a highly virulent strain of *Pseudomonas solanacearum* from Mexico using stem-inoculation with 5.6x10^-1^ to 1.2x10^8^ CFU/plant which resulted in 50% wilted plants at an inoculum of 3-100 CFU/plant and 2.1x10^6^ CFU/plant in a more resistant clone of *S. phureja.* However, invasive infection *via* stem-inoculation may overestimate the disease response and cannot simulate natural exposure of the pathogen through contaminated irrigation water. Montanelli et al. (1995) inoculated potato plants by non-invasive root inoculation to differentiate between resistant and susceptible cultivars. The authors concluded that a dose of 5x10^6^ CFU/plant was necessary to identify resistant plants. However, infections were still observed in all three cultivars at the lowest tested concentration of 5x10^5^ CFU/plant. To our knowledge, only one study by Singh et al. (2014) investigated the effect of low concentrations (10^1^-10^10^ CFU/mL) of *R. solanacearum* on potted tomato plants in a greenhouse setting. In their experiments, a single irrigation event containing a dose of 5x10^3^ CFU of *R. solanacearum* resulted in bacterial wilt incidence. At an inoculum of 5x10^2^ CFU, infections were only observed when roots had been injured before irrigation.

As part of a quantitative microbial risk assessment, we investigated the dose-response relationship between *R. solanacearum* and potted potato in greenhouse experiments. In addition, the minimum concentration required under optimal conditions for disease development was assessed in experiments with *in vitro* plants using cultivars with different pathogen susceptibility. Dose-response models are essential in risk assessments and have already been applied in studies related to human health and pathogen exposure after the consumption of treated drinking water (Schijven et al., 2019). Dose-response models in the context of plant health are scarce (Boelema, 1985) or used to evaluate the efficacy of biological controls for plant pathogens (Montesinos and Bonaterra, 1996; Smith et al., 1997). The aim was to develop a dose-response model that eventually can be applied to value the effectivity of (irrigation) water treatment systems that remove pathogens and provide safe irrigation water.

## 2 Material and methods

### 2.1 Bacterial strain and growth conditions

*R. solanacearum* phylotype II (race 3 biovar 2) strain IPO-4187 was received from the working collection at Wageningen UR and used in this study. Strain IPO-4187 was derived by making *R. solanacearum* strain PD2762, isolated from potatoes in the Netherlands in 2016, rifampicin resistant following the protocol described by van der Wolf et al. (2022). The strain belongs to sequevar 1 (R. solanacearum phylotype IIB-1) (Anonymous, 2018). The strain was kept at -80°C using the multi-purpose protect cryobeads system (Technical Services Ltd, Lancashire, GB) and revived on casamino-acid-peptone-glucose (CPG) agar before the experiment (Hendrick and Sequeira, 1984). CPG is composed of 1 g/L casamino acids, 10 g/L peptone, 5 g/L glucose and 15 g/L agar. Inoculation suspensions were prepared from cultures grown overnight in CPG broth at 28°C while shaking (150 rpm). Cultures were harvested by centrifugation (3500 x *g*, 20 min at room temperature) followed by washing and resuspending the pellet in a quarter strength Ringer’s solution (in the following referred to as “Ringer’s solution”; Sigma-Aldrich; St. Louis, USA). This pelleting and washing step was repeated twice to remove any excess broth. Lastly, Ringer’s solution was added to resuspend the pellet and the bacterial suspension was then diluted to reach an optical density of 0.1 at 600 nm representing a concentration of approximately 10^8^ CFU/mL which was confirmed by dilution-plating. *Ralstonia solanacearum* was detected on the semi-selective medium South Africa (SMSA) (Elphinstone et al., 1998) supplemented with 50 mg/L rifampicin to suppress the growth of background bacteria and 200 mg/L cycloheximide to suppress fungal growth. Duchefa Biochemie (Haarlem, NL), Sigma-Aldrich (St. Louis, MO, USA), and Fisher Scientific (Hanover Park, IL, USA) were our chemical suppliers.

### 2.2 Greenhouse experiments

#### 2.2.1 Plants and growth conditions

During the summers of 2019 and 2020, the dose-response relationship of *R. solanacearum* and potato (*Solanum tuberosum*) was assessed in a climate controlled compartment of the Unifarm greenhouse facilities of Wageningen University & Research, Wageningen (NL). Certified pest-free minitubers of cultivar (cv) Kondor (2019) and HB (2020) were obtained from Agrico (Emmeloord, NL) and stored at 4°C. Both cultivars are susceptible for *R. solanacearum* but there is no further information available how resistant these strains are towards the pathogen. For the experiment, 100 mini-tubers were presprouted in the light for about two weeks at 15°C until they were planted in 5 L plant pots containing 4 kg clay loam obtained in spring 2019 from an agricultural field of Unifarm which was prepared for potato cultivation. The soil consisted of about 27% clay, 33% sand and 32% silt. Half of the soil was used for the experiment in 2019 while the rest was stored at 4°C for the experiment in 2020. The air dried soil was roughly sieved (1 x 1 cm mesh size) before filling the pots. In each pot, an about 10 cm deep hole was dug in the soil where one mini-tuber was placed inside with the sprouts put up and covered with a few centimeters of soil. Pots were placed on saucers and the soil was watered from above. Temperature of the greenhouse was set to 23°C and 70% humidity during the experiment. After emergence of the plants, a 16-h light period was achieved using supplemental lighting (high-pressure sodium lamps, 150 W/m^2^) when needed. Till inoculation of the potato plants, the soil was daily watered from above. Once a week fluid nutrient solution (Yara Netherlands, Vlaardingen, NL) for potted tomato plants was applied following the manufacturer’s guidelines. Five days before inoculation, plants received restricted watering. After inoculation, the plants were watered *via* the saucers to avoid cross-contamination caused by splashing or formation of aerosols. To avoid water saturation of the soil of bacterial wilt diseased plants, the irrigation volume was adapted to the plant’s needs. The plants were inspected weekly for symptom expression till the end of the experiments.

In addition to the potato plants, 6 (2019) or 10 (2020) tomato plants cv Moneymaker were grown in the same greenhouse compartment to check the virulence of the *R. solanacearum* inoculum used in the potato dose-response experiments. Watering was done the same way as with the potato plants. The tomato plants were inspected weekly for symptom expression during four weeks.

Simultaneous with the 2020 dose-response experiment, the die-off of a *R. solanacearum* population was studied in soil without the presence of a host plant. The experiment was done in two sets, each consisting of a control and three soil contaminated pots, respectively. One set (disturbed) was used for sampling of the soil 7, 14, 21, 28, 35 and 54 days post inoculation (dpi). The other set was sampled only at the end of the experiment (undisturbed) to exclude influence of the soil sampling on the pathogen’s persistence. The control pot of the undisturbed set was used to monitor the soil moisture, temperature and humidity during the experiment (Hobo Soil Moisture Smart Sensor connected to a data logger, Onset Computer Corporation, MA, USA). Pots (5 L) filled with 4 kg of the same soil as used in the dose-response experiment were used. The soil of the *R. solanacearum* population die-off was watered from above during the length of the experiment as no plant roots were present to suck the water from the saucer.

#### 2.2.2 Selection of inoculation doses

The goal was to simulate natural infection of potato plants by *R. solanacearum* using a single irrigation event with contaminated water. Several factors were considered in the selection of doses. First, we used irrigation data from a potato growing field in the North of the Netherlands (53.2945693 N, 7.0045595 E). There, the drip irrigation doses for potato cropping were monitored for three consecutive years during the cropping season (2016-2018) and ranged between 53 and 176 mm (Acacia Water, 2019). The highest irrigation dose was supplied during the dry summer in 2018. Secondly, we considered *R. solanacearum* concentrations in contaminated surface water from different regions in the Netherlands which were up to 10^2^ CFU/mL during the summer months (Wenneker et al., 1999). In a conservative scenario, the maximum dose applied to a potato plant during the cropping season would result in about 7x10^5^ CFU; calculated from 176 mm irrigation, a soil surface in the pot of 415 cm^2^, and the concentration of *R. solanacearum* in contaminated surface water (10^2^ CFU/mL). Based on this dose, we selected a range of doses from 5x10^2^ to 5x10^9^ CFU per pot as shown in **Table 1A**.

Table 1Setup for dose-response experiments in greenhouse (A) and *in vitro* (B) for potato cultivars Kondor and HB, where potato plants were inoculated with selected concentrations of *Ralstonia solanacearum*.

**Table:** 

A. Greenhouse experiments										
Number of plants per cultivar		5	15	15	15	15	10	10	10	5
Inoculation concentration^1^	CFU/mL	control	10^1^	10^2^	10^3^	10^4^	10^5^	10^6^	10^7^	10^8^
Estimated dose	CFU	control	5x10^2^	5x10^3^	5x10^4^	5x10^5^	5x10^6^	5x10^7^	5x10^8^	5x10^9^
Dose per g soil (4 kg soil)	CFU/g	control	0.13	1.3	13	1.3x10^2^	1.3x10^3^	1.3x10^4^	1.3x10^5^	1.3x10^6^
Dose per soil surface area^2^	CFU/cm^2^	control	1.2	12	1.2x10^2^	1.2x10^3^	1.2x10^4^	1.2x10^5^	1.2x10^6^	1.2x10^7^

**Table:** 

B. *In vitro* experiments										
Nr. of plants per repetition and cultivar (2 repetitions)		5	15	15	15	15	10	10	10	
Inoculation concentration^3^	CFU/mL	control	5x10^2^	5x10^3^	5x10^4^	5x10^5^	5x10^6^	5x10^7^	5x10^8^	
Estimated dose	CFU	control	0.5	5	50	500	5x10^3^	5x10^4^	5x10^5^	

^1^50 mL applied for non-invasive soil-soak inoculation; ^2^soil pot surface area = 415 cm^2^; ^3^1 µL applied on stem wounding.

#### 2.2.3 Inoculation procedure

Five days before inoculation, plants received restricted watering. In the dose-response experiments, unwounded potato plants (36 days old in 2019, 30 days old in 2020) were inoculated *via* a soil-soak inoculation assay (Tans-Kersten et al., 2001) by pouring 50 mL of *R. solanacearum* suspension with the respective concentration onto the soil around the stem, followed by watering with 200 mL water to let the inoculum penetrate into the soil. The pot surface area was 415 cm^2^, therefore, the plants were irrigated with a total of 6 mm of which 1.2 mm (50 mL) were the inoculation suspension. Of the 100 plants grown for each experiment 95 plants were inoculated with bacterial suspension. Five plants treated with 50 mL Ringer’s solution served as negative controls (**Table 1A**). As we expected few infections in the lower inoculum concentrations (10^1^ – 10^4^ CFU/mL), 15 plants were inoculated per concentration while 10 plants per concentration were inoculated with the higher concentrations (10^5^ – 10^7^ CFU/mL), and 5 plants with the highest concentration of 10^8^ CFU/mL. After inoculation, the plants were sorted in 5 replicate blocks of 20 plants in which each dose was represented proportionally (e.g. 1 control plant per block or 3 plants of the dose 5x10^2^ CFU).

In the experiment in which the die-off of *R. solanacearum* in soil was studied without plants, soil was contaminated with the pathogen following the same procedure as was used in the dose-response experiment. Per pot, 50 mL of a 10^8^ CFU/mL bacterial suspension was applied onto the soil corresponding to 1.3x10^6^ CFU/g. The controls were treated with Ringer’s solution.

Three (2019) or five (2020) tomato plants were inoculated by injecting ca. 25 µL of a suspension of 10^8^ CFU/mL of *R. solanacearum via* the leaf axil of the 2^nd^ or 3^rd^ true leaf into the stem. The same number of plants were used as control, respectively, and injected with Ringer’s solution.

#### 2.2.4 Disease scoring

The potato plants were monitored weekly after inoculation by visual inspection for typical bacterial wilt symptoms. The monitoring period was 38 dpi for cv Kondor and 54 dpi for cv HB. Per plant, the total amount of stems was counted and each stem was scored individually using the following categories: 0 – no wilting; 1 – 0-25% of stem wilted; 2 – 26-50% of stem wilted; 3 – 51-75% of stem wilted; 4 – 76-100% of stem wilted. Per plant and time point a disease index (DI) was calculated:


$$DI=\frac{\sum^{}wilting\;scores\;per\;stem\;\;}{total\;number\;of\;stems}$$

Finally, a mean DI value was calculated considering the total number of plants for each treatment and their respective DIs. Healthy plants were also included in the calculation of total number of stems.

#### 2.2.5 Plant and soil sampling

At the end of the dose-response experiments, plant material (stems, tubers and roots) and a mixed soil sample consisting of bulk and rhizosphere soil were collected per plant to test for the presence of *R. solanacearum*.

Segments of all stems per asymptomatic plant were collected by cutting 4-5 cm long pieces ca. 5 cm above the soil and placed without preceding surface sterilization in an universal extraction bag (Bioreba Ag, Reinach, CH). For symptomatic plants, only the stems showing symptoms were collected. Stem samples were further processed on the day of sampling as described below. Stem segments of tomato plants which served as pathogenicity check for *R. solanacearum* were collected in the same way and processed without preceding surface sterilization. After collecting the stem samples, the plant pot was turned upside down in a clean plastic tub of which the inside had been disinfected with 70% EtOH and covered with a clean paper towel. Then, the different subterranean plant plants and soil were carefully separated to take samples.

Tubers were removed by hand from the stolons and placed into a paper bag. Adhering soil on the tubers was not removed to avoid damaging of the peel. The paper bags were stored max. 1-2 weeks inside a storage box at room temperature together with silica gel to remove moisture and avoid rotting during storage. Before further processing, tubers were gently wiped with paper tissue to remove dried adhering soil. The peel at the stolon end was removed with a disinfected (70% EtOH) knife and the vascular tissue lying below spooned out, placed in a zip-lock extraction bag (4x6 cm, 0.09 mm thick LDPE, unbranded) and processed as described below.

Roots were subsequently cut off from the stem base with disinfected (70% EtOH) scissors, separated from the bulk soil and vigorously shaken in two subsequent batches of 250 mL tap water to remove attached soil. In cv Kondor, only the roots of non-symptomatic plants were analyzed and as positive control, two roots samples of symptomatic plants inoculated with 5x10^8^ CFU. The washed roots were dabbed dry on tissue paper and stored overnight in a plastic bag at room temperature till surface sterilization. Before surface sterilization, the root sample was transferred to a 180 mL screw-cap plastic container (Corning, Gosselin, NY, USA). Then, 0.01% Tween 80 solution was poured over the roots and gently agitated by hand for 5 min to soak off attached soil particles and fatty components. After draining off the Tween 80 solution, the roots were covered with 1: 4 diluted household bleach (active reagent sodium hypochlorite ca. 5%) and gently agitated for another 5 min. Finally, the surface sterilized roots were washed four times with tap water, before being transferred into a zip-lock extraction bag (8x6 cm, 0.09 mm thick LDPE, unbranded) and processed as described below. After removal of the plant parts, the remaining soil was thoroughly mixed by hand. Randomly selected soil samples were transferred with a disinfected (70% EtOH) spoon to a zip-lock plastic bag till a total of ca. 30 g soil was collected. Excess air was pushed out before closing the bag. Soil was stored at room temperature till being further processed as described below.

The top of the soil in the pots of the disturbed set of the *R. solanacearum* population development experiment was sampled weekly and the monitoring period was 53 days after soil contamination. Per pot, soil was collected from three randomly selected locations in the upper soil layer by digging a 5 cm deep hole with a disinfected (70% EtOH) spoon which was carefully closed again after sampling. Then, the collected soil was mixed and about 6 g composite soil sample was transferred to a 15 mL falcon tube and directly processed as described below.

#### 2.2.6 Sample processing

Samples of plant material in extraction bags were weighed, and then macerated by crushing at 2 bar pressure for about 20-30 sec in a Sample crusher (AAA Lab Equipment, Roelofarendsveen, NL). Then Ringer’s solution (buffer volume equivalent to twice the weight of the plant material) was mixed through the crushed material for about 5 minutes to allow bacteria to diffuse out of the tissues into the fluidal phase. To soil samples, a volume of Ringer’s solution equivalent to twice the weight of the soil material was added, followed by vigorous vortexing for 1-2 min. Plant macerates were spread-plated undiluted on SMSA supplemented with 200 mg/L cycloheximide and 50 mg/L rifampicin. Additionally, serial dilutions of soil suspensions were prepared (undiluted, 10x and 100x diluted in Ringer’s solution) and also spread-plated in duplicates. After incubation for 3-5 days at 25°C, plates were inspected for the presence of *Ralstonia*-like colonies. Colonies from the soil samples of the *R. solanacearum* soil population development experiment were enumerated. Although the SMSA supplemented with rifampicin suppressed successfully the growth of non-target bacteria after plating suspensions from plant material, some non-target bacteria grew when plating the soil samples. To identify *R. solanacearum*, all *Ralstonia*-like colonies re-isolated from plant or soil material were checked with colony-PCR. To do so, a *Ralstonia*-like colony was selected from the plate and suspended in 50 µL de-ionized water in a PCR reaction tube where it was boiled for 10 min at 95°C using a PCR cycler, after which it could be stored at -20°C. To identify the *R. solanacearum* by PCR, we followed the protocol of Schönfeld et al. (2003) which amplifies a *fliC* fragment (5’- GAA CGC CAA CGG TGC GAA CT-3’; 3’- GGCGGCCTTCAGGGAGGTC-5’) and results in a 400 bp PCR product that was visualized on a 1% agarose gel. To the PCR reaction mix, 100 nm of the forward and reverse primer were given together with 5 µL of the boiled colony.

### 2.3 *In vitro* experiments

Using stem-inoculated potato *in vitro* plants, we investigated the effect of very low doses of *R. solanacearum* on its host under conditions considered optimal for multiplication of the pathogen and disease development. *In vitro* plants of *S. tuberosum* cv Kondor and HB were obtained from Agrico (Emmeloord, NL). Plants were propagated in clear polypropylene tissue culture vessels (Duchefa Biochemie, Haarlem, NL) filled with Murashige & Skoog (MS) agar. This medium consisted of 4.4 g/L MS salts, 30 g/L sucrose and 7 g/L plant agar, with a final pH of 5.8. *In vitro* plants were maintained in a light incubator at 25°C and 16 h photo period. For the experiment, only the top part of the shoot of each plant was propagated to obtain a uniform sample. One shoot was placed in a ‘De Wit’ culture tube filled with 7 mL MS (Duchefa Biochemie, Haarlem, NL). The experiment was executed in two independent replicates using both potato cultivars and 80 plants per cultivar and repetition. In total, 320 plants were tested and **Table 1B** shows the overview of the experimental setup. Before inoculation, the cut shoots were grown for about 50 days. Plants were inoculated by firstly wounding the stem of the plants with a sterile needle (0.8 mm diameter, B. Braun Melsungen AG, Germany) ca. 2 cm above the agar. Secondly, 1 µL of bacterial suspension with the respective concentration was pipetted on the wound. The droplet was absorbed within about 15 minutes by the plant, after which the tubes were closed and wrapped with cling film. Disease symptoms were monitored after 10 and 17 dpi using the same disease scoring scale as in the greenhouse experiments. To detect latent infections, plants were analyzed for the presence of *R. solanacearum* by re-isolating the bacterium from stem tissue in the same way as the greenhouse experiments. In short, plants were cut with a sterile knife 1-2 cm above the inoculation point, placed in a plastic zip-lock extraction bag (4x6 cm, 0.09 mm thick LDPE, unbranded), macerated and suspended in 0.5 mL of Ringer’s solution. Suspensions were enumerated in Tryptone Soya Agar (TSA; Oxoid, Basingstoke, UK) supplemented with 50 mg/L rifampicin using the pour plating method in 24-well plates. For the pour plating, autoclaved TSA was maintained liquid at 50°C and supplemented with rifampicin. Plant extract (100 µL, undiluted, 10x, 100x, 1000x diluted) was added in duplicates into 24-well plates, after which 300 µL of the liquefied TSA+rifampicin medium were added to each well while shaking steadily to allow mixing before the agar solidified. As control, 100 µL of Ringer’s solution was added to the medium. Plates were incubated at 25°C for 3-4 days until bacterial colonies within the medium were counted using a binocular.

### 2.4 Dose-response model

The response of a host after being challenged with a pathogen can be described in two steps (Teunis and Havelaar, 2000; Teunis et al., 2018). At first, the pathogen has to successfully enter the host, overcoming its natural defense barriers and multiply to infect the plant. Assuming that every pathogen has the same fixed probability *p_m_* to enter the host, overcome its barriers (*p_1_, …, p_m_*) and cause a disease, the dose-response relationship for infection is described as an exponential dose response model:


(1) $$P_{inf}\left(cV|p_{m}\right)=1-\;e^{-p_{m}cV}$$

where *p_m_* quantifies the host-pathogen interaction, *c* is the pathogen concentration in a certain volume *V*, where *cV* represents the exposure dose, the number of pathogens.

However, it is more realistic that the interaction between host and pathogen is heterogeneous and not constant. Instead of using a fixed probability of infection described by *p_m,_* the infection is described with the infection parameters *α* and *β.* In this case, the host-pathogen interaction follows a Beta probability distribution, which can be described by the Beta-Poisson model for microbial infection. *α* and *β* are the infection parameters and _1_ *F*_1_ is the confluent hypergeometric function (Teunis and Havelaar, 2000):


(2) $$P_{inf}\left(cV|\alpha ,\beta \right)=1{-}_{1}F_{1}\;\left(\alpha ,\alpha +\beta ;\;-cV\right)$$

*α* and *β* are Monte Carlo sample pairs (joint distribution) reflecting uncertainty and variability of infectivity. They were transformed to improve parameter estimation:


(3) $$u_{1}=\;\frac{\alpha }{\alpha +\beta };\;\;w_{1}=log\left(\frac{u_{1}}{1-u_{1}}\right)$$

$$v_{1}=\;\alpha +\beta ;\;z_{1}=log\left(v_{1}\right)\;\;\;\;$$

*w*_1_ is thereby a measure of infectivity (location) and *z*_1_ is a measure of variation in infectivity (spread).

In a second step, the conditional probability of illness (symptomatic plants) within the group of infected plants is described with the hazard model of illness dose response.


(4) $$P_{ill|inf\;}\left(cV\right)=1-\;{\left(1+\frac{cV}{\eta }\right)}^{-r}$$

Similarly to infection, illness parameters *r* and *η* were transformed into location parameters *w*_2_ and *z*_2_:


(5) $$u_{2}=\;\frac{r}{r+\eta };\;\;w_{2}=log\left(\frac{u_{2}}{1-u_{2}}\right)$$

$$v_{2}=\;r+\eta ;\;z_{2}=log\left(v_{2}\right)\;\;\;\;$$

Log-likelihood ratio (LR) testing was applied to select the best dose-response model for infection, exponential (Eq. 1) or Beta-Poisson (Eq. 2) (Teunis et al., 1996). LR tests were also used to compare individual dose-response datasets (e.g. stem infection) with pooled datasets (all observations of infections of different plant parts are combined: stem, root or tuber infection). Also, we compared the dose-response models of both cultivars to test whether a pooling of these datasets was admissible. Therefore, values for parameters p_m_ or α and β that maximize the log-likelihood function were estimated. The log-likelihood function is described as:


(6) $$\ell \;\left(\alpha ,\;\beta \right)=-2\sum_{i=1}^{k}\left\{I_{i}\text{log }P_{inf}\left(D_{i}\right)+\;\left(T_{i}-I_{i}\right)\;\log(1-P_{inf\;}\left(D_{i}\right)\right\}$$

where ℓ is the likelihood, k the number of doses, T_i_ the number of plants exposed a certain dose of which I_i_ were infected. *P_inf_* represents the values obtained from Eq. (1) or Eq. (2) with *D_i_* = c* *V_i_*. The parameter values that optimize this function ( $\hat{l}$ ) are $\hat{p_{m}}$ , $\hat{\alpha }$ and $\hat{\beta }$ .

The best fit of the dose-response relation given a specific dataset is achieved with the maximum likelihood estimates $\hat{\alpha }$ and $\hat{\beta }$ . The likelihood supremum without any constraints is calculated as followed:


(7) $$\ell_{sup}=\sum_{i=1}^{i=n}\;\left[I_{i}\log\left(\frac{I_{i}}{T_{i}}\right)+\;\left(T_{i}-I_{i}\right)\;\log\left(\frac{T_{i}-I_{i}}{T_{i}}\right)\right]$$

The model was written and run in JAGS (Just Another Gibbs Sampler, v4.3.0) (Plummer, 2015) from R (v4.1.2) (R Core Team, 2022) to assess uncertainty. JAGS is a system for Markov chain Monte Carlo (MCMC) sampling for Bayesian hierarchical models. The source code of the JAGS model and LR testing is provided in S1. For each Monte Carlo simulations, 3 chains were run in parallel; after a burn-in of 1000 iterations, the model was run for 10^5^ iterations. Wide priors were used for the parameters, and their influence on the posterior predictive samples was checked by using different means and variances for *w* and *z*. Prior values together with the source code can be found in S1.

### 2.5 Die-off model for soil population development

The die-off of *R. solanacearum* populations in non-cultivated soil was modelled with a non-linear Weibull + tail model as described in Eisfeld et al. (2021).


(8) $$C_{t}=\;(C_{0}-\;C_{res})\;e^{-{\left(\alpha t\right)}^{\beta }}+\;C_{res}$$

where *C_t_* is the bacterial concentration [M/L^3^] at time *t* [T], *C_0_* the initial bacterial concentration [M/L^3^] (at time *t* = 0), *C_res_* [M/L^3^] is the residual bacterial population at the end of the observation period. *α* [1/T] is a scale parameter and *β* [-] a shaping parameter to display convexity of a curve if 0< *β<* 1, or simulates a shoulder effect when *β* > 1. If *C_res_* = 0, a non-linear Weibull model is obtained. Plus, the model can be reduced to a log-linear die-off model if *β* = 1. The Weibull + tail, the Weibull and the log-linear model can be compared using the AIC (Akaike Information Criterion). The model with the best fit has the lowest AIC. Note that the inoculation concentration at t = 0 was 1.3x10^6^ CFU/g but the first sample was taken 7 dpi to allow an adaptation of the bacterium to the soil environment and not disturb by sampling.

## 3 Results

### 3.1 Dose-response relationship in the greenhouse

**Figure 1:**
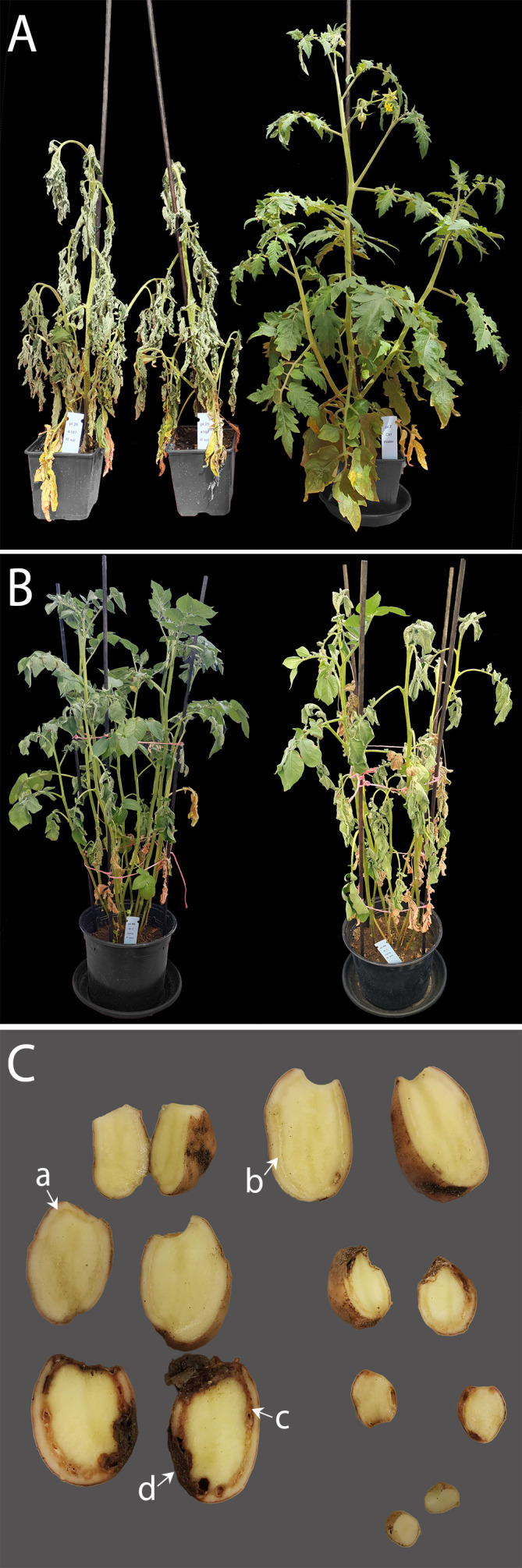
**(A)** Tomato cv Moneymaker were stem-inoculated (10^8^ CFU**)** to confirm the virulence of *Ralstonia solanacearum* 4187; two inoculated plants next to a non-inoculated control plant (right). **(B)** Bacterial wilt in potato cv Kondor after soil-soak inoculation with 5x10^8^ CFU, left plant shows symptoms 24 dpi and right plant 35 dpi; **(C)** Progeny tubers of a potato plant inoculated with 5x10^9^ CFU; stolon ends have been removed for analysis; tubers show typical infection symptoms of *R. solanacearum* in different stages of infection indicated by the arrows – the vascular ring discolors (a) and white bacterial ooze emerges (b) followed by browning of the vascular ring (c) and rotting of tuber tissue (d).

In two greenhouse experiments, the disease response of potted potato plants to *R. solanacearum* was monitored for 38 and 54 days using cultivars Kondor and HB, respectively. Plants were inoculated by irrigation with contaminated water containing different concentrations of the bacterium. Tomato plants cv Moneymaker were stem inoculated (5x10^8^ CFU) during both experiments. The tomato plants showed wilting mild symptoms after 7 dpi with a mean disease index of 2. At 14 dpi, all plants showed heavy wilting symptoms (DI = 4) which confirmed the virulence of the strain (see **Figure 1A**). The potato cultivars differed in their growth characteristics as cv HB developed very long stems and grew to about two times the height as cv Kondor (**Figure S1**). The tuber yield was lower in cv HB than in cv Kondor. While a potato plant of cv Kondor had on average nine progeny tubers with an average weight of 170 g, cv HB had on average two progeny tubers with an average weight of 57 g (**Table S1**).

#### 3.1.1 Disease development

**Figure 2:**
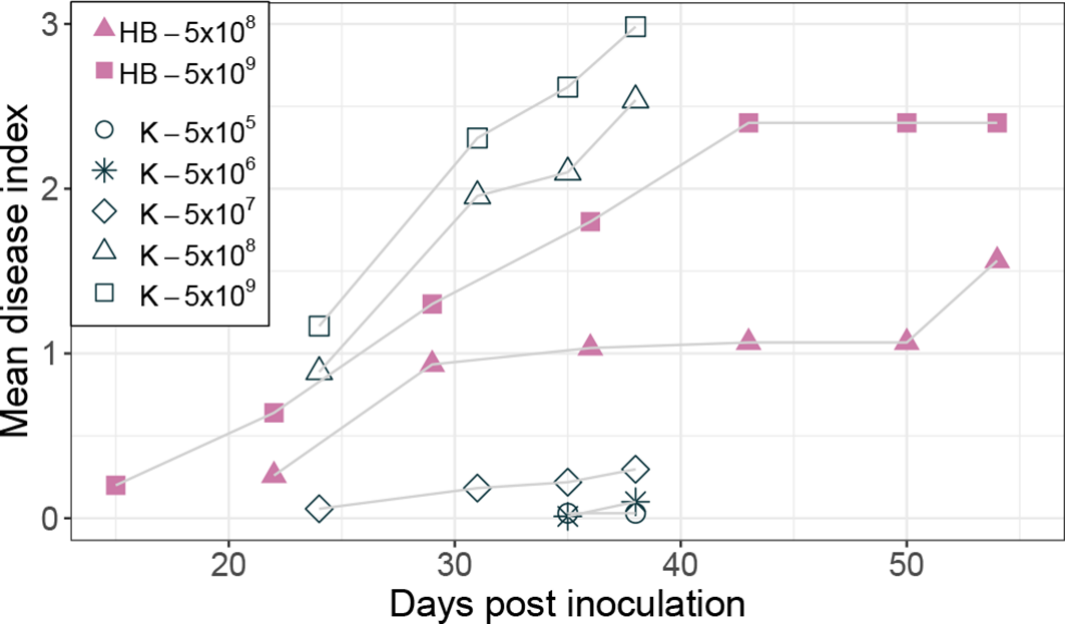
Development of bacterial wilt disease of potato plants cv Kondor (K, open black symbols) and HB (filled purple symbols) in greenhouse experiments inoculated with different doses of *Ralstonia solanacearum* in CFU, as indicated in the legend. The mean disease index was assessed by scoring the wilting symptoms of stems on a scale from 0 (no wilt) – 4 (100% wilted). The score of no wilt (0) is not displayed in the graph.

Inoculation of potatoes with high bacterial numbers (5x10^8^ and 5x10^9^ CFU) resulted in fast disease symptom development as shown in **Figure 1B**. **Figure 2** shows the development of the disease progress by scoring wilting symptoms of the stems and calculating the mean DI for each treatment over time. First wilting symptoms were observed in plants inoculated with 5x10^9^ CFU at 15 dpi in cv HB and at 24 dpi in cv Kondor exposed to the same dose. In the experiment with cv Kondor, some plants (also controls) were affected by yellowing and brown spots of unknown cause. This may have hampered the clear identification of early stages of *R. solanacearum* infections, but overall it was possible to distinguish between the browning and wilting symptoms. In the end, 19 plants of cv Kondor with wilting symptoms were observed after inoculation with 5x10^5^ – 5x10^9^ CFU (**Table 2**). At a lower dose of 5x10^5^ CFU or 5x10^6^ CFU, only 1 of the 15 or 10 plants showed symptoms, respectively. This resulted in a maximum mean DI of 3 at 5x10^9^ CFU and a minimum DI of 0.03 at 5x10^5^ CFU. Only seven plants of cv HB showed bacterial wilt symptoms at the two highest inoculation concentrations. For both cultivars, wilting increased steadily until about 40 dpi if the plants had been treated with a higher dose (**Figure 2**). Thereafter, the disease progress slowed down in cv HB which was monitored longer (about 20 days) than cv Kondor. This resulted in a plateau phase from 43 to 54 dpi. In this plateau phase, all symptomatic plants of cv HB inoculated with 5x10^8^ CFU or 5x10^9^ CFU became 100% wilted and the mean DI did not increase anymore. Only at the last observation point (54 dpi), a newly wilted plant that had been treated with 5x10^8^ CFU, was observed which resulted again in an increase of the mean DI. In some plants, very fast disease development was observed. For example, one plant of cv HB inoculated with 5x10^8^ CFU did not show any symptoms at 22 dpi but thereafter all four stems wilted 100% within one week (29 dpi). Overall, we observed more wilting and infections in cv Kondor than in cv HB and symptoms were only observed at a dose higher than 5x10^5^ CFU. At the highest dose, 100% of the plants of cv Kondor were wilted and 50% plants of cv HB.

**Table 2:** Symptomatic (wilting) and non-symptomatic infections of potted potato plants cv Kondor and HB after a single non-invasive soil soak inoculation with different doses of *Ralstonia solanacearum*.

	Dose (CFU)		Ctrl	5x10^2^	5x10^3^	5x10^4^	5x10^5^	5x10^6^	5x10^7^	5x10^8^	5x10^9^
	Nr. of exposed plants		5	15	15	15	15	10	10	10	5
cv Kondor	Nr. of plant parts infected with *R. solanacearum* ^1^	stem	0	1	2	0	2	3	5	10	5
		root	0	1	0	0	0	0/9	5/9	2/2	–
		tuber	0	0	0	0	0	0	4	8/8	4/4
		soil	0	3	1	0	5	1	4	10	5
	Nr. of infected plants^2,3^		0	1	2	0	2	3	5	10	5
	Nr. of plants showing wilting symptoms (illness)^3^		0	0	0	0	1	1	2	10	5
	Nr. of latently infected plants^4^		0	1	2	0	1	2	3	–	–
cv HB	Nr. of plant parts infected with *R. solanacearum* ^1^	stem	0	0	0	0	0	0	1	4	3
		root	0	0	1	0	1	0	0	3	2
		tuber	0	0	0	0	0	0	0	1/5	0/1
		soil	0	0	0	0	0	0	0	4	5
	Nr. of infected plants^2,3^		0	0	1	0	1	0	1	4	3
	Nr. of plants showing wilting symptoms (illness)^3^		0	0	0	0	0	0	0	4	3
	Nr of latently infected plants^4^		0	0	1	0	1	0	1	0	0

^1^Based on SMSA dilution plating and confirmation by colony-PCR (when two numbers are separated by a slash the first number represents the number of infected parts and the second number the number of samples tested); ^2^any plant part infected (stem, root or tuber); ^3^these numbers were used as input infection or illness data in the dose-response (DR) model; ^4^plants infected with *R. solanacearum* but without wilting symptoms.

#### 3.1.2 Analysis of plant material

At the end of the experiment, different plant parts (stem, root or tuber) were analyzed for *R. solanacearum.* Thereby, the presence of *R. solanacearum* in symptomatic plants was confirmed and latent infections (infected plants not showing wilting symptoms) were found (**Table 2**). In total, nine plants of cv Kondor were latently infected mostly in the stems and roots of plants exposed to a low dose (5x10^2^ or 5x10^3^ CFU). In the experiment with cv HB, three plants with latent infections were observed of which two of them in the roots, inoculated with 5x10^3^ or 5x10^5^ CFU. In some of the tubers produced by the plants, infections were already so advanced that disease symptoms were visible on the tuber skin (browning around the stolon) or inside when cutting the tubers in half (**Figure 1C**). Only at a high dose of at least 5x10^7^ CFU infected progeny tubers were found in both cultivars. Moreover, some symptomatic plants of cv Kondor treated with a high dose did not produce progeny tubers, or tubers were already rotten at the end of the experiment and could not be further analyzed. At the end of the experiments, from each plant, a mixed sample of bulk of rhizosphere soil was analyzed. For cv Kondor, soil remained contaminated with *R. solanacearum* at all tested concentrations except for 5x10^4^ CFU while for cv HB the soil samples were found positive only at the two highest inoculation concentrations.

#### 3.1.3 Dose-response model

**Figure 3:**
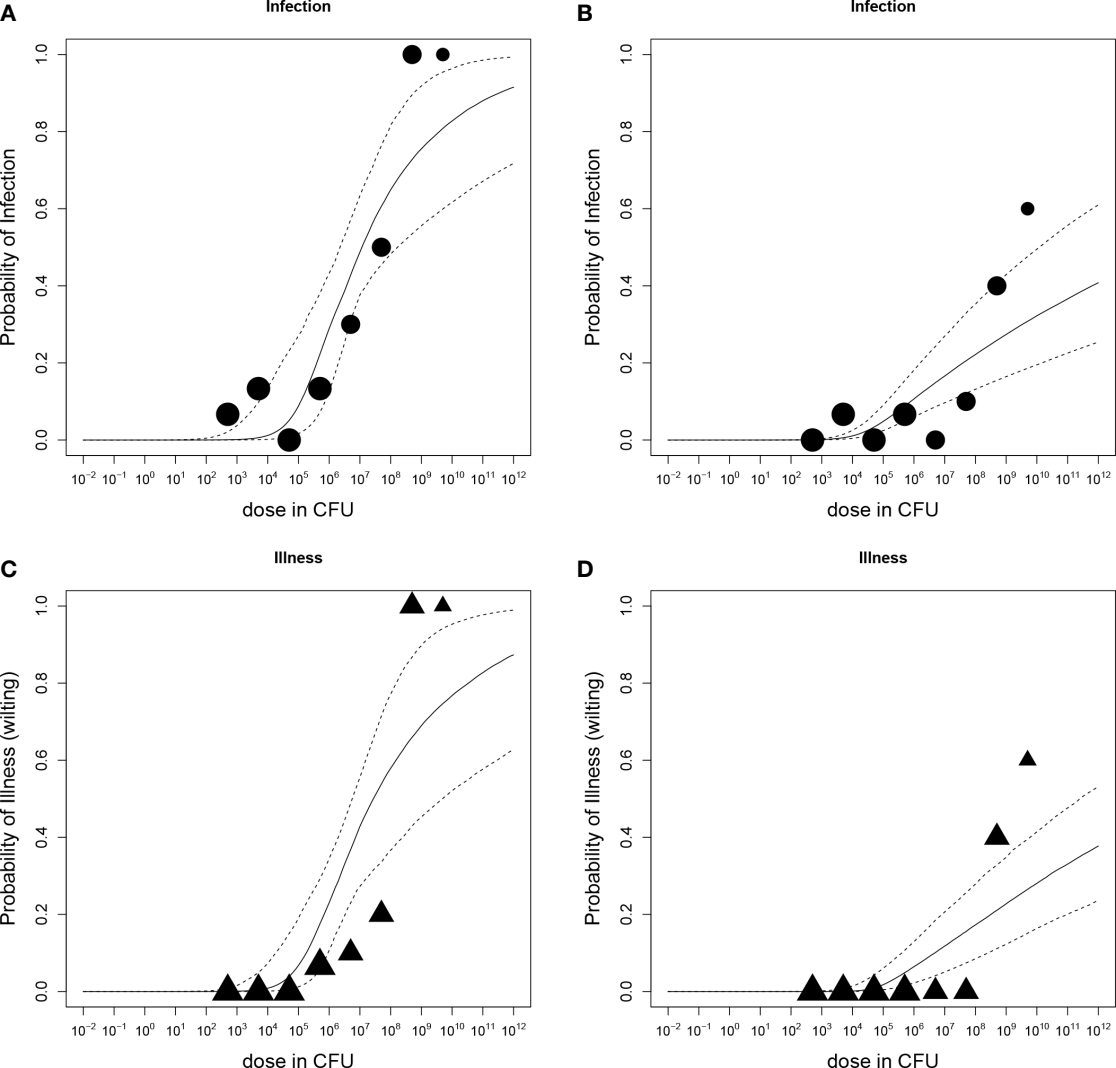
Dose-response relations of potato plants cv Kondor **(A, C)** or cv HB **(B, D)** and *Ralstonia solanacearum*; A+B: Probability of infection (stem, root, or tuber) with increasing dose in colony forming units (CFU); **(C, D)** Probability of illness (wilting) with increasing dose. Each graph shows the median and 95% range of the probability of infection or illness, indicated by the dashed line, calculated by the dose-response model as a function of dose. Available data are shown as a bubble chart in which the symbol size is proportional to the number of plants challenged.

Dose-response relations for *R. solanacearum* infections of potato plants are shown in **Figure 3** and estimated infection and illness parameters are presented in **Table 3**. For the dose-response model infections of all plant parts (stem, root or tuber) at the end of the experiments were considered. A plant that was found positively infected in two different plant parts (e.g. stem and root) was only counted once (**Table 2**). The Beta-Poisson model was a better model than the exponential model according to the LR test. The dose-response model indicated a higher probability of infection or illness of cv Kondor by *R. solanacearum* than of cv HB. While the dose-response relation showed a similar trend for both cultivars at lower inoculation concentrations (e.g. 10% infection probability at a dose of 1.3x10^6^ CFU in cv Kondor and 7.2x10^6^ CFU in cv HB, the model is steeper for cv Kondor indicating higher probability of infection at a higher inoculation concentration. The ED_50_ (dose required to infect or wilt 50% of the plants) of infection was 1.1x10^7^ CFU for cv Kondor. Ninety percent of the plants of cv Kondor were predicted to be infected at a dose of 2.7x10^12^ CFU, whereas at the same dose for cv HB only 40% of the plants are predicted to get infected. The dose-response models for both cultivars predicted that there exists a 0.3% infection probability at a dose of about 2x10^4^ CFU. The illness dose-response model was similar to the infection dose-response model for both cultivars. Nevertheless, less symptomatic plants were observed at low doses in comparison to high inoculation doses.

Table 3Estimated parameters of dose-response models for infection and illness and respective values of the 95% confidence interval.

**Table:** 

			Infection						Illness (symptomatic plants)						
Greenhouse experiments															
potato cv	DR model	**α**	2.5%	97.5%	**β**	2.5%	97.5%	**ED_50_**	**r**	2.5%	97.5%	**η**	2.5%	97.5%	**ED_50_**
Kondor	bp	0.17	0.07	0.38	3.7x10^5^	1.6x10^3^	2.2x10^6^	1.1x10^7^	1.6x10^5^	0.04	6.2x10^3^	4.5 x10^5^	3.1x10^-5^	8.4x10^4^	2.7x10^7^
HB	bp	0.03	0.02	0.05	2.6x10^4^	8.8x10^3^	6.1x10^4^	*1.2x10^10^	4.7x10^2^	0.05	4.9x10^3^	2.6 x10^4^	7.6x10^3^	6.1x10^4^	*2.5x10^11^

**Table:** 

*In vitro* experiments															
pooled Kondor + HB	bp	0.46	0.11	0.89	0.13	6.0x10^-3^	0.41	0.90	1.4x10^4^	0.19	4.8x10^3^	9.4Ex10^2^	1.1x10^-5^	1.1x10^2^	1.13

DR, dose-response; cv, cultivar; bp, Beta-Poisson; ED_50_, dose required to infect or wilt 50% of the plants;

*value taken from upper limit of 95% confidence interval.

### 3.2 Dose-response relationship under *in vitro* conditions

**Figure 4:**
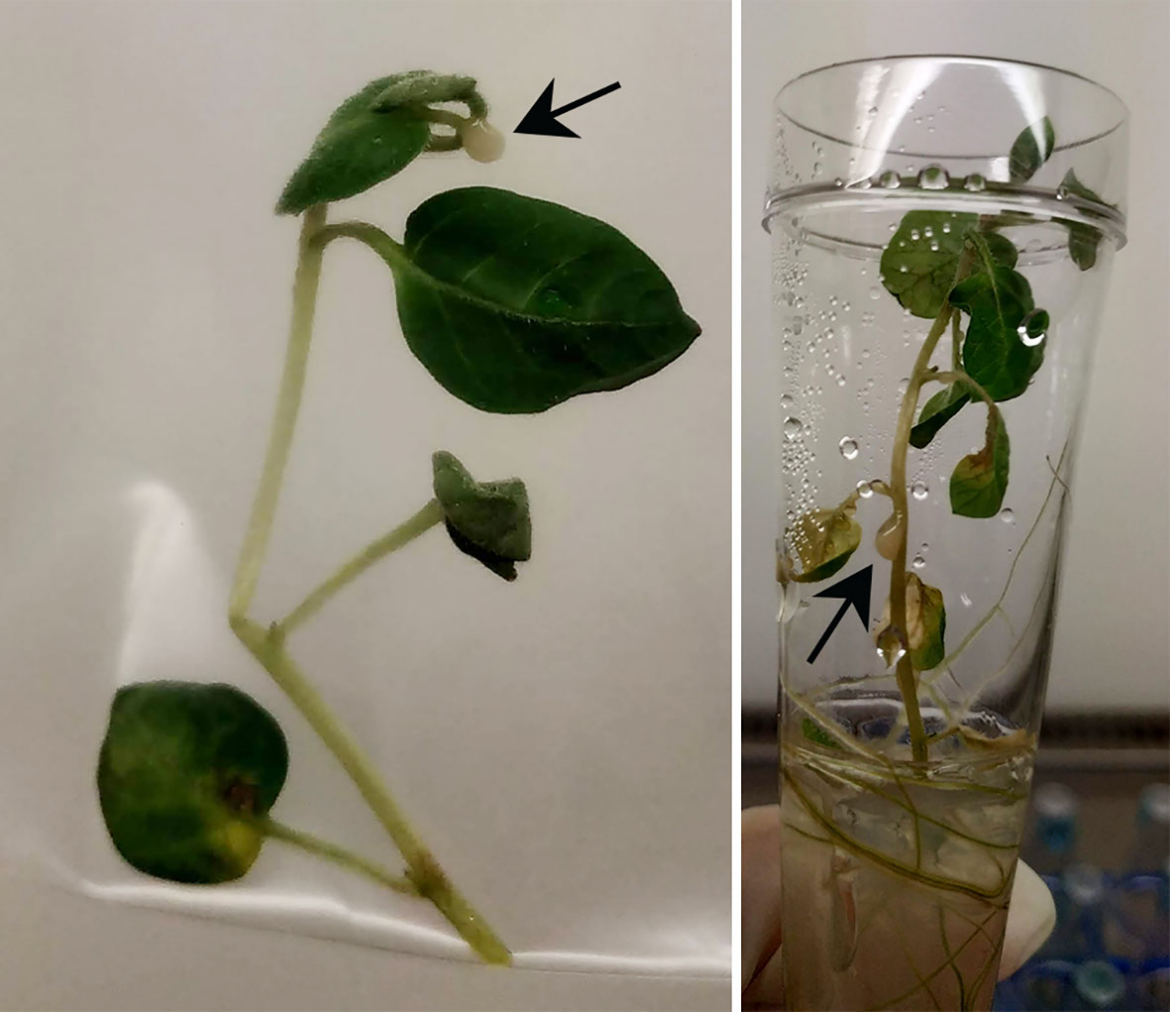
*In vitro* plants infected with *Ralstonia solanacearum* at 15 dpi; Arrows indicate oozing of bacterial slime, containing high concentrations of bacteria.

Both potato cultivars were tested under sterile *in vitro* conditions to evaluate the dose response under conditions considered optimal for multiplication of *R. solanacearum* and disease expression. In contrast to the greenhouse experiments, the stems of the potato plants were wounded first, followed by placing 1 µL of bacterial suspension on the wound. This resulted in faster and more severe disease responses (**Table 4**). Some of the plants even showed oozing of bacterial slime from the stem containing a high concentration of *R. solanacearum* (**Figure 4**). First wilting was recorded at 10 dpi in all higher inoculation concentrations (>100 CFU). Six wilted plants of cv HB (replicate 1) were found after inoculation with 50 CFU and one wilted plant after inoculation with 5 CFU in cv Kondor. Another week later at 17 dpi, all plants of both cultivars in both replicates showed heavy wilting symptoms and even at an inoculation dose of 0.5 CFU about 20-30% of plants were wilted (**Table 4**). The ED_50_ for infection of *in vitro* plants was as low as 0.90 CFU. After the observation period, plants were harvested and analyzed for the presence of *R. solanacearum* which led to the detection of a few (2-3) latently infected plants at lower inoculum of 0.5 and 5 CFU. Nearly all plants inoculated with higher inoculum were symptomatic after two weeks.

**Table 4:** Symptomatic (wilting) and non-symptomatic infections after stem-inoculation of *in vitro* potato plants of two cultivars Kondor and HB with different doses of *Ralstonia solanacearum*.

	Dose [CFU]		0	0.5	5	50	500	5x10^3^	5x10^4^	5x10^5^
	Nr. of exposed plants		10	30	30	30	30	10	10	10
cv Kondor	Nr. of plants showing wilting symptoms	10 dpi	0	0	2	1	12	7	10	6
		17 dpi	0	10	21	28	29	10	10	10
	Nr. of infected plants^a^	17 dpi	0	10	27	29	30	not tested	not tested	not tested
cv HB	Nr. of plants showing wilting symptoms	10 dpi	0	0	0	6	15	10	10	10
		17 dpi	0	8	25	28	30	10	10	10
	Nr. of infected plants^a^	17 dpi	0	10	28	29	30	not tested	not tested	not tested

a re-isolation of *R. solanacearum* from stem material; dpi, days post inoculation.

**Figure 5:**
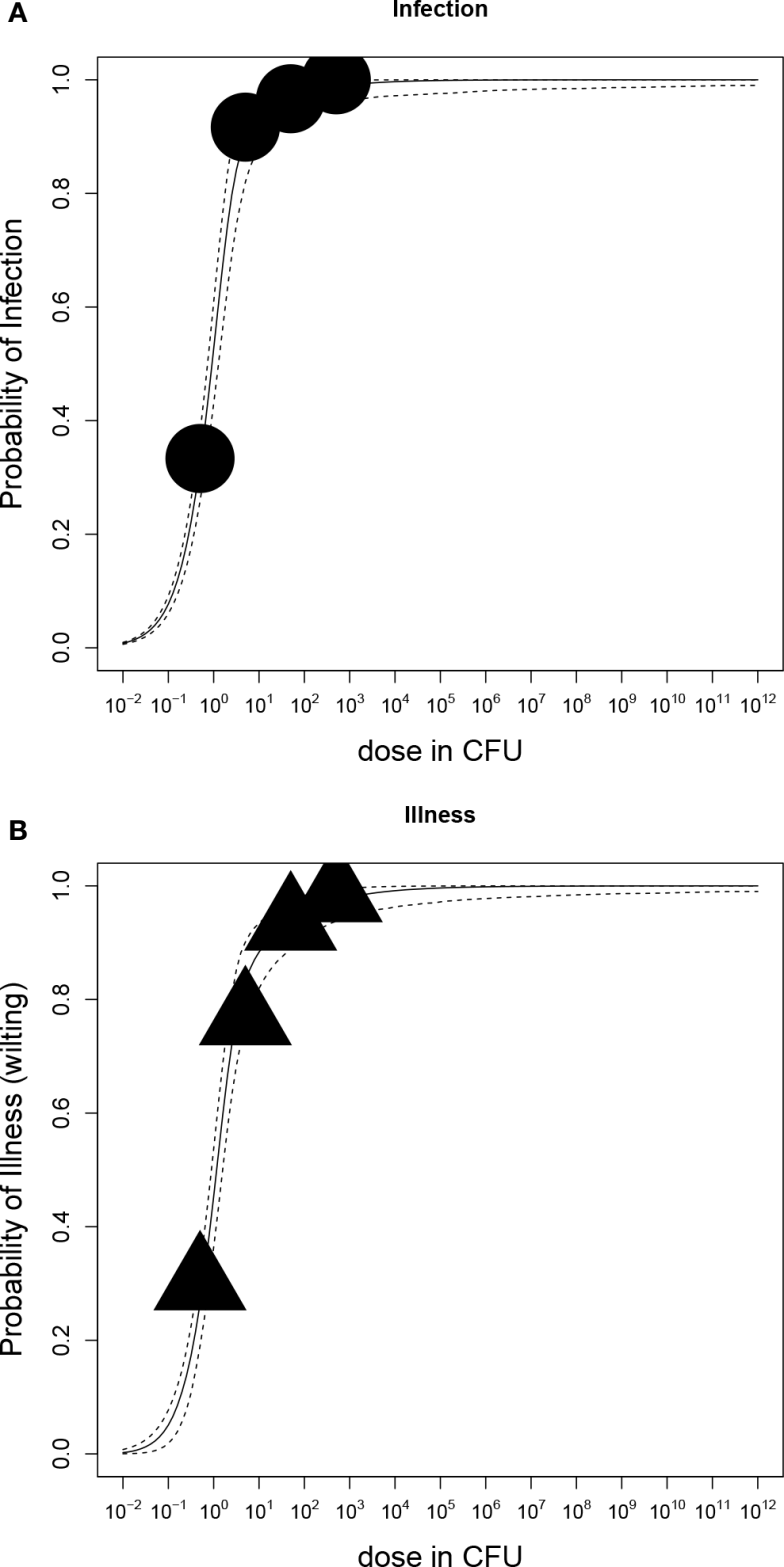
Dose-response relations of potato plants grown *in vitro* and *Ralstonia solanacearum*); **(A)** Probability of stem-infection with increasing dose in colony forming units (CFU); **(B)** Probability of illness (symptomatic plants) with increasing dose. Each graph shows the median and 95% range of the probability of infection or illness, indicated by the dashed line, calculated by the dose-response model as a function of dose. Available data are shown as a bubble chart (symbol size proportional to the number of plants challenged).

The dose-response relation of *in vitro* potato plants and *R. solanacearum* is shown in **Figure 5** and corresponding parameter estimates are presented in **Table 3**. According to the LR test, all datasets of cultivars Kondor and HB could be pooled and the Beta-Poisson model was the better model than the exponential. In comparison to the greenhouse experiments the dose-response graph of *in vitro* experiments is shifted to the left as a much lower dose already caused infection or illness. As in the greenhouse experiments, the dose-response model for illness and infection were similar, indicating that the probability of an infection at a given dose has the same probability to result in illness.

### 3.3 Die-off in infested soil

**Figure 6:**
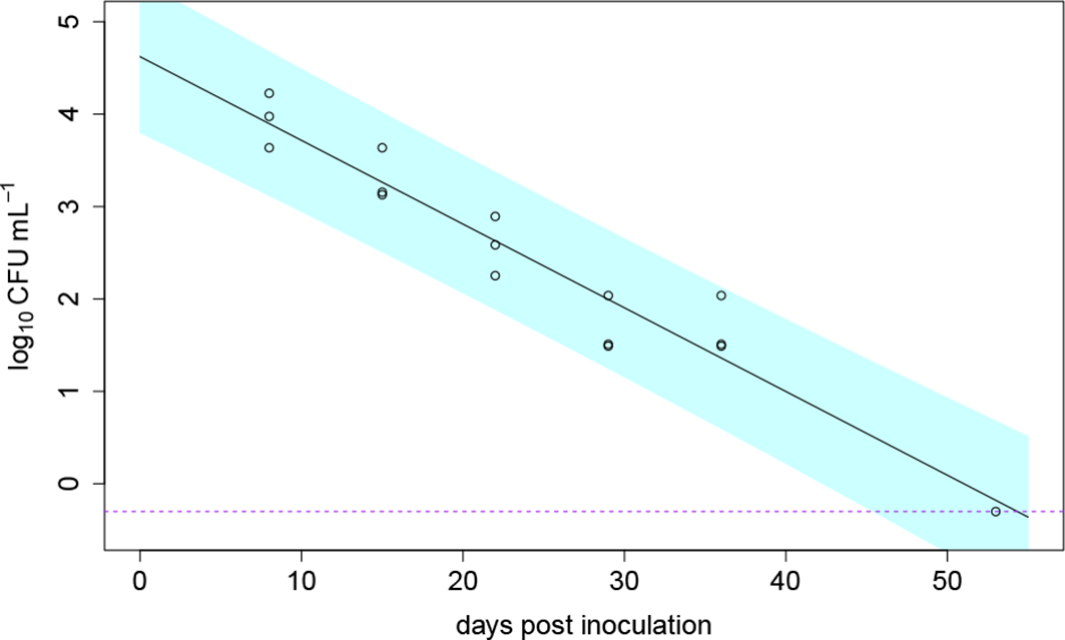
Die-off of *Ralstonia solanacearum* over time in in three pots filled with clay loam soil without the presence of a host plant; the data shown as open circle symbols were fitted with a log-linear die-off model (solid line); the dashed purple line indicates the detection limit and the blue band indicates the 95% prediction interval; at the last measurement point 54 days post inoculation, the bacterium was only detectable in one of the three pots.

The die-off of a soil population of *R. solanacearum* without the presence of a plant was monitored simultaneously to the greenhouse experiments in 2020. At the end of the experiment (54 dpi), *R. solanacearum* was detectable in one of three inoculated pots which were sampled regularly (disturbed set of pots). The die-off of *R. solanacearum* in soil without the presence of a host plant was modelled using a non-linear Weibull model. Best model fit (comparing the AIC) was achieved using the log-linear die-off model *C_t_* = *C*_0_ *e* ^–^ *^αt^*. The die-off in soil is shown in **Figure 6**. The predicted model parameters resulted in a C_0_ of 10^4^ CFU/g (± 1.5) and a die-off rate of α = 0.09 (± 0.007) 1/day. At 54 dpi, the undisturbed set of pots (soil analyzed only at 54 dpi) were analyzed for the bacterium. There, *R. solanacearum* was only detected in one of the three inoculated pots which was similar to the results of the disturbed pots.

## 4 Discussion

In potato production, water is crucial to support plant growth and tuber production. Due to decreasing availability of fresh water of adequate quality, water treatment schemes are required to provide irrigation water and secure food production. However, plant pathogens such as *R. solanacearum* may not be fully removed during the treatment and pose a risk spreading plant diseases. This is the first study to investigate the dose-response relationship between *R. solanacearum* and potato plants grown in pots in a greenhouse setting, simulating infection through irrigation with contaminated water at low concentrations. The presented dose-response model can be used in quantitative microbial risk assessment (QMRA) to evaluate water treatment systems on their pathogen removal capacity.

At first, we found that the two tested potato cultivars showed different susceptibility to *R. solanacearum* under greenhouse conditions. Inoculation of cv Kondor with the two highest doses (5x10^8^ and 5x10^9^ CFU) resulted in 100% infection of all plants, whereas only 50% of plants cv HB were infected when inoculated with the same doses. Susceptibility of a cultivar is determined by its genetic traits and breeding for resistance is an important tool to control bacterial wilt although so far no resistant potato cultivar exists (Huet, 2014). Although cv HB showed a higher resistance against *R. solanacearum*, it had less favorable agronomic traits because it produces very long stems and less progeny tubers than cv Kondor. As a result, cultivation of cv HB has been discontinued by the breeding company. Additionally, although partially resistant or tolerant cultivars can be desirable in countries with a high level of endemic infections, they may also contribute to an unnoticed multiplication of latently infected plant material and a spread of the pathogen. In this way, partially resistant or tolerant cultivars may even contribute to the build-up of *R. solanacearum* infections over time (Swanson et al., 2005).

In both cultivars, latent infections were discovered after maceration and analysis of the plant material. Studying the locations of infection within the plant allowed to retrace the infection pathway of *R. solanacearum*. According to literature, after the soil-soak inoculation, the bacterium first enters the plant through root openings followed by a systemic colonization of the xylem vessels of the stems (Siddiqui et al., 2014). All root-infected plants of cv Kondor were also stem infected indicating fast and successful colonization. Plants of cv HB inoculated with 5x10^3^ and 5x10^5^ CFU were only infected in their roots indicating an early infection stage. It is not clear if these root infections would have developed into a systemic colonization after the time span of the experiment. Note that not the whole root system of each plant was analyzed. Therefore, it is possible that some root infections remained undetected. Consequently, sampling of roots can be useful for detecting early infections but may underestimate the actual infection incidence as processing of the complete root system is laborious. Our experiments demonstrated that latent infections also occurred at lower inoculation concentrations in stem and root although to a very low extent. The infected plants may develop symptoms when observed over a longer time period as the dose-response model predicts the same probability for infection or illness after contact with a given dose. Latent infections of propagation material contribute to the spread of plant diseases. Nevertheless, infected progeny tubers were only found at high inoculum densities (>10^7^ CFU) but not when irrigated with low doses. This is an important outcome as trading of latently infected seed tubers poses a high risk spreading brown rot disease internationally (EFSA et al., 2019).

While the greenhouse experiments simulated irrigation with contaminated water under natural conditions, *in vitro* experiments allowed to study the dose-response relationship under conditions (high temperature, availability of water and nutrients, absence of competition by other micro-organisms) expected to be optimal for the multiplication of *R. solanacearum* and for disease expression at very low inoculation concentrations. Moreover, stem-inoculation is more accurate in applying a certain dose where the bacterium is brought in direct contact with the plant. Soil-soak inoculation simulates natural contamination but not all bacteria may have reached and invaded the plant. As a result, even the lowest dose of 0.5 CFU caused infections under *in vitro* conditions using stem-inoculation. Note that we assumed the bacterial inoculation suspension to be Poisson distributed where on average 0.5 CFU should be present in 1 µL, but the actual doses might have been higher or lower than 0.5 CFU because of the random distribution of the bacterial cells in the inoculation solution (Haas et al., 2014). Eventually, the invasive inoculation method by stem injury promoted disease development even at the lowest dose. Singh et al. (2014) showed that injuring the roots of tomato plants (grown in a greenhouse in pots with autoclaved soil mixture) increased wilt disease. In their experiments, an inoculum level of 10^2^ CFU/mL resulted in 63% wilting when roots were injured, while plants with uninjured roots inoculated with 10^3^ CFU/mL resulted in only about 7% of diseased plants. In their experiments no wilted plants were observed when inoculated with 10 CFU/mL regardless of root wounding. In contrast, in our greenhouse experiments, latently infected plants (stem and root) were found after inoculation with minimally 10 CFU/mL (dose of 5x10^2^ CFU). However, symptoms were only observed at an inoculum level of 10^4^ CFU/mL (dose of 5x10^5^ CFU) in our experiments. Consequently, the information gained from the *in vitro* experiments showed a precise dose-response relationship but it should be used with caution. It shows that under these conditions, fast and severe disease expression (wilting and oozing) may occur. But the *in vitro* results may not be useful to estimate the dose-response relationship under field conditions as most external influences on the host-pathogen system are excluded. Moreover, the *in vitro* experiments did not reproduce the different susceptibilities of the two tested potato cultivars as their disease progress was similar. In the *in vitro* experiments, the bacteria were directly introduced into the vascular system of the plant by stem injection. The pathogen did not have to recognize, attach and penetrate the plant to overcome its external barriers when colonizing the plant during infection (Huet, 2014). In the field, bacterial infections are facilitated by wounds which are caused by feeding insects on the stem or root nematodes, or by natural openings where secondary roots will emerge (Champoiseau et al., 2009). In such a case, the dose-response model obtained from *in vitro* experiments may be adequate to estimate the high infection risk of potato plants by *R. solanacearum*. In the greenhouse experiments, however, root openings may have also been present in potato plants with growing roots. Plus, the natural soil from an agricultural field still contained organisms that may have damaged the root surface. Habe (2018) presented an *in vitro* assay that allowed to differentiate resistances between bacteria. In their study, *in vitro* plants were grown in sterile vermiculite with MS liquid medium and soil-inoculated with >10^2^ CFU/mL, but lower doses as in our study have not been tested. The use of *in vitro* plants may be an effective way to study the pathogen virulence as symptom development occurred already at a very low dose of even a single cell. At present, pathogenicity has to be tested on a host under greenhouse conditions (Anonymous, 2018). The *in vitro* assay offers a less laborious and less expensive alternative to the standard pathogenicity test.

Currently, a zero tolerance policy in Europe prohibits the use of surface water in which *R. solanacearum* has been detected for irrigation of potato crops (Anonymous, 1998). Our dose-response results indicate that this legislation is appropriate if high concentrations of *R. solanacearum* (e.g. released from wild host plants) are present in surface water. However, *R. solanacearum* will generally be found at lower concentrations in surface waters of maximum 10^3^ CFU/mL (Wenneker et al., 1999; Álvarez et al., 2007) which only have a very low probability of infection. Moreover, water treatment like natural sand filtration can reduce bacterial concentrations by several log_10_ to improve water quality which decreases the risk spreading brown rot using treated irrigation water (Eisfeld et al., 2022). As with drinking water, irrigation water quality regarding microbiological safety can be analyzed using QMRA (Anonymous, 2011). In this context, dose-response models are an important component of QMRA to evaluate risks related to water reuse in irrigation.

Finally, practical aspects of potato production should be considered when applying dose-response models. First, irrigation water quality is not only important during crop cultivation but also before planting. Farmers may need to irrigate their soil to guarantee sufficient soil moisture which promotes root development and seed germination (Letnes, 1958). Therefore, we also analyzed the persistence of *R. solanacearum* after soil irrigation without a potato plant present where the bacterium persisted for up to 54 dpi when inoculated with 1.3x10^6^ CFU/g. In comparison, soils of the greenhouse experiments where plants were irrigated with high doses, similar to the soil die-off experiment, all soils remained contaminated with *R. solanacearum*. Therefore, our results indicate that the presence of a potato plant prolonged the pathogen’s soil persistence even though the plant was not infected (e.g. cv Kondor treated with 10^2^ CFU). Plants release root exudates and nutrients which attract the bacteria by chemotaxis towards the root surface (Yao and Allen, 2006). Consequently, persisting pathogen populations in the soil introduced *via* contaminated irrigation water can infect the planted seed tubers (Messiha et al., 2007). Second, the irrigation method may influence the dose-response results. This study analyzed soil-soak inoculation to simulate drip irrigation where the bacteria can enter the plant *via* the roots. In overhead irrigation, also the leave surface gets in contact with the irrigation water which may result in a different dose-response model. Third, the irrigation frequency should be considered as continuous irrigation with low contaminated water may result in a build-up of the pathogen in the soil from where it can infect the plant. Lastly, research should explore the dose-response relationships with other hosts and plant pathogens. Our research showed that variability exists even between the same host-pathogen system if different host cultivars are used as potato cultivars depict different susceptibilities to brown rot (Bowman and Sequeira, 1982; Montanelli et al., 1995). In a risk assessment, we recommend applying the dose-response model of cv Kondor as it had a higher infection probability which will deliver more conservative risk estimates.

To conclude, due to increasing water scarcity in agriculture, water reuse schemes will gain more importance and regulations should consider water treatment as an effective way to reduce plant pathogens. Although a 100% removal can never be guaranteed, QMRA in combination with dose-response models can analyze the effectiveness of water treatment (Schijven et al., 2011; Verbyla et al., 2016). Dose-response models are an essential element of risk analysis as they allow to translate pathogen exposure into risk of infection which has a direct practical implications. If exposure after a water treatment remains too high, modifications or additional treatments can be included in order to minimize the pathogen related risks and provide sufficient water quality and quantity in agriculture.

## Data availability statement

The original contributions presented in the study are included in the article/**Supplementary Material**. Further inquiries can be directed to the corresponding author.

## Author contributions

CE designed the experiment, collected, analyzed and interpreted the data and drafted the original version of the article. JS helped with the experimental design, data analysis and modeling, and critical review of the manuscript. PK helped with the experimental design and execution of experiments including regular inspection of the plants, and critical review of the manuscript. BB conceived the project and funding, gave input for the experimental setup and critically reviewed the manuscript. GM conceived the project and funding and contributed to the experimental design. JV conceived the project and funding. PT helped with the data analysis and especially the coding for the dose-response model and critical review of the manuscript. JW helped with the experimental design, data interpretation, critical review of the manuscript. All authors contributed to the article and approved the submitted version.
