# Supplementary material for: Dose-response relationship of Ralstonia solanacearum and potato in greenhouse and in vitro experiments
Source: Front Plant Sci. 2022 Dec 20;13:1074192. doi: 10.3389/fpls.2022.1074192 (PMC10020725; doi:10.3389/fpls.2022.1074192)
Supplement: Supplementary file 1 [file DataSheet_1.docx]

Supplementary Material

# S1: Code to run the dose-response model

The model was written and run in JAGS (v4.3.0) (Plummer, 2015) from R (v4.1.2) (R Core Team, 2022) to assess uncertainty. JAGS is a system for Markov chain Monte Carlo (MCMC) sampling for Bayesian hierarchical models. For each Monte Carlo simulations, 3 chains were run in parallel; after a burn-in of 1000 iterations, the model was run for 10^5^ iterations. Wide priors were used for the parameters, and their influence on the posterior predictive samples was checked by using different means and variances for *w* and *z*.

## Source code in JAGS

### Exponential model

model {

# parent nodes: hyperparameters

# status: infection =1; illness = 2

for(status in 1:2){

w[status] ~ dnorm(mu.w[status], 1/si.w[status]^2);

z[status] ~ dnorm(mu.z[status], 1/si.z[status]^2);

u[status] <- exp(w[status]) / (1+exp(w[status]));

v[status] <- exp(z[status]);

a[status] <- u[status]*v[status];

b[status] <- (1-u[status])*v[status];

}

for(ind in 1:n.doses) {

# infection

prinf[ind] <- 1 - exp(-u[1]*exp(logdose[ind]));

infec[ind] ~ dbin(prinf[ind],expos[ind]);

# symptoms

prill[ind] <- (1-pow(1+(exp(logdose[ind])/b[2]),-a[2]));

sympt[ind] ~ dbin(prinf[ind]*prill[ind],expos[ind]);

}

#

} # end model

### Beta-Poisson model

model {

# parent nodes: hyperparameters

# status: infection =1; illness = 2

for(status in 1:2){

w[status] ~ dnorm(mu.w[status], 1/si.w[status]^2);

z[status] ~ dnorm(mu.z[status], 1/si.z[status]^2);

u[status] <- exp(w[status]) / (1+exp(w[status]));

v[status] <- exp(z[status]);

a[status] <- u[status]*v[status];

b[status] <- (1-u[status])*v[status];

}

for(ind in 1:n.doses) {

# infection: low dose

prexp[ind] <- 1- exp(-exp(logdose[ind]))

pbeta[ind] <- u[1];

ld.prinf[ind] <- prexp[ind]*(pbeta[ind]);

# infection: high dose

num[ind] ~ dpois(exp(logdose[ind]));

gamma[ind] <- loggam(a[1]+b[1]) - loggam(a[1]+b[1]+num[ind]) +

loggam(b[1]+num[ind]) - loggam(b[1]);

hd.prinf[ind] <- (1-exp(gamma[ind]));

prinf[ind] <- ifelse(logdose[ind] < 0,ld.prinf[ind],hd.prinf[ind]);

infec[ind] ~ dbin(prinf[ind],expos[ind]);

# symptoms

prill[ind] <- (1-pow(1+(exp(logdose[ind])/b[2]),-a[2]));

sympt[ind] ~ dbin(prinf[ind]*prill[ind],expos[ind]);

}

#

} # end model

## Source code in R

### Input data

potato.2019 <- list(

exp = c(15,15,15,15,10,10,10,5),

dose= c(5e2,5e3,5e4,5e5,5e6,5e7,5e8,5e9),

ill = c(0,0,0,1,1,2,10,5), # 2019

inf = c(1,2,0,2,3,5,10,5));

potato.2020 <- list(

dose= c(5e2,5e3,5e4,5e5,5e6,5e7,5e8,5e9),

exp = c(15,15,15,15,10,10,10,5),

ill = c(0,0,0,0,0,0,4,3), # 2020

inf = c(0,1,0,1,0,1,4,3));

potato.iv <- list( # in vitro results of Kondor and HB

dose= c(0.5,5,50,505),

ill = c(18,46,56,59),

exp = c(60,60,60,60),

inf = c(20,55,58,60));

# libraries and working directory

# and create output folder where results are stored

library(rjags) – alternative: library(runjags) with updated functionalities, not used here

library(boot)

library(writexl)

library(gsl)

setwd("C:/Users/**xxx**/") # adapt to your own computer

### MCMC sampling and JAGS model

# - define monte carlo markov chain (MCMC) parameters

# - name of output files

# - access JAGS model via R, stored in same folder as working direction as JAGS file:

**# here, R will access the JAGS model (section 1.1)**

nburn <- 1000;

niter <- 100000;

thin <- 1;

tomonitor <- c("w","z","a","b");

potato.iv <- list(

dose= c(0.5,5,50,505),

ill = c(18,46,56,59),

exp = c(60,60,60,60),

inf = c(20,55,58,60));

data <- potato.iv

# define file names of all output files and location

file.mod.exp <- paste(ver,".model_exp.","jags",sep="");

file.res <- paste("./output/",ver,".result.","pdf",sep="");

file.xtr <- paste(ver,".extract.","r",sep="");

file.drs <- paste(ver,".doseresp.","r",sep="");

file.txt <- paste("./output/",ver,".result.","txt",sep="");

file.dat <- paste(ver,".data.","r",sep="");

file.pst <- paste("./output/",ver,".post.","rda",sep="");

file.par <- paste("./output/",ver,".mcmc.","rda",sep="");

make.jags.data <- function(data,priors){

logdose <- log(data$dose);

n.doses <- length(logdose);

return(list("mu.w"=priors$mu.w, "si.w"=priors$si.w,

"mu.z"=priors$mu.z, "si.z"=priors$si.z,

"logdose"=logdose, "expos"=data$exp,

"infec"=data$inf, "sympt"=data$ill,

"n.doses"=n.doses));

}

# definition of priors

wzpriors <-list(

mu.w = c(-20,-10), # mean of w for infection, illness

si.w = c(5,5), # sd of w for infection, illness

mu.z = c(10,10), # mean of z for infection, illness

si.z = c(5,5)); # sd of z for infection, illness

drdata <- make.jags.data(potato.iv,wzpriors);

cat("<<< Compile model >>>\n");

mod.pst <- jags.model(file=file.mod.exp,data=drdata,n.chains=3);

update(mod.pst,n.burn=nburn);

cat("<<< MCMC Sampling >>>\n");

mcmc.pst <- coda.samples(mod.pst,tomonitor,n.iter=niter,thin=thin);

cat("<<< Store results >>>\n");

sink(file.txt);

print(summary(mcmc.pst));

sink();

cat("<<< Graphing results >>>\n");

pdf(file.res);

plot(mcmc.pst,trace=TRUE,density=FALSE);

plot(mcmc.pst,trace=FALSE,density=TRUE);

dev.off();

cat("<<< Store posterior >>>\n");

save(mcmc.pst,file=file.pst,ascii=TRUE);

### Beta-Poisson model

## define dataset and priors

dir.create(file.path(getwd(), "output_bp")) # create new output folder

data <- potato.2019 # alternative: potato.2020 or potato.iv

# definition of priors for potato.2019

wzpriors <-list(

mu.w = c(0,0), # mean of w for infection, illness

si.w = c(5,5), # sd of w for infection, illness

mu.z = c(0,0), # mean of z for infection, illness

si.z = c(5,5)); # sd of z for infection, illness

# Choose this for low infectivity: potato.tuber; potato.stem

# mu.w << 0 for low infectivity

# mu.z >> 0 for steep slope (near exponential shape)

# si.z small for small variability in slope

# But better still: exponential model!!!

# choose for potato.2020

# wzpriors <-list(

# mu.w = c(-10,-10), # mean of w for infection, illness

# si.w = c(5,5), # sd of w for infection, illness

# mu.z = c(10,10), # mean of z for infection, illness

# si.z = c(0.5,0.5)); # sd of z for infection, illness

### MCMC sampling and JAGS model

**# here, R will access the JAGS model (section 1.1.2)**

nburn <- 1000;

niter <- 100000;

thin <- 1;

ver <- "dr";

tomonitor <- c("w","z","a","b");

# tomonitor <- c("a","b");

make.jags.data <- function(data,priors){

logdose <- log(data$dose);

n.doses <- length(logdose);

return(list("mu.w"=priors$mu.w, "si.w"=priors$si.w,

"mu.z"=priors$mu.z, "si.z"=priors$si.z,

"logdose"=logdose, "expos"=data$exp,

"infec"=data$inf, "sympt"=data$ill,

"n.doses"=n.doses));

}

drdata <- make.jags.data(potato.2019,wzpriors);

cat("<<< Compile model >>>\n");

mod.pst <- jags.model(file=file.mod,data=drdata,n.chains=3);

update(mod.pst,n.burn=nburn);

cat("<<< MCMC Sampling >>>\n");

mcmc.pst <- coda.samples(mod.pst,tomonitor,n.iter=niter,thin=thin);

cat("<<< Store results >>>\n");

sink(file.txt);

print(summary(mcmc.pst));

sink();

cat("<<< Graphing results >>>\n");

pdf(file.res);

plot(mcmc.pst,trace=TRUE,density=FALSE);

plot(mcmc.pst,trace=FALSE,density=TRUE);

dev.off();

cat("<<< Store posterior >>>\n");

save(mcmc.pst,file=file.pst,ascii=TRUE);

cat("<<< Store parameter estimates >>>\n");

cat("<<< Contour plot of w-z space >>>\n");

cat("<<< Dose response graphs >>>\n");

### **Likelihood analysis of microbial dose response**

# - compare the infection dose-response curves of different plant material. e.g. compare infection curve of stem with infection curve of roots

# - are they significantly different?

# - does the data follow exponential of Beta-Poisson dose-response relationship

# - can data be pooled?

# define a few infection dose response models

drinf<- function(a,b,dose) dr1f1(a,b,dose)

drexp <- function(pm,dose) 1-exp(-dose*pm)

# define the illness dose response model

drill <- function(a,b,dose) 1-(1+dose/b)^(-a)

likbp.inf <- function(data,ab){

p <- rep(NA,length(data$dose));

for(k in 1:length(data$dose)) p[k] <- drinf(ab[1],ab[2],data$dose[k])

lik <- (p^data$inf)*((1-p)^(data$exp-data$inf));

return(prod(lik));

}

likxp.inf <- function(data,pm){

p <- rep(NA,length(data$dose));

for(k in 1:length(data$dose)) p[k] <- drexp(pm,data$dose[k])

lik <- (p^data$inf)*((1-p)^(data$exp-data$inf));

return(prod(lik));

}

lik.cond.ill <- function(data,ab){

p <- rep(NA,length(data$inf));

for(k in 1:length(data$inf)) p[k] <- drill(ab[1],ab[2],data$dose[k])

lik <- (p^data$ill)*((1-p)^(data$inf-data$ill));

return(prod(lik));

}

to.wz <- function(ab){

u <- ab[1]/(ab[1]+ab[2]);

v <- ab[1] + ab[2];

return(c(log(u/(1-u)),log(v)));

}

to.ab <- function(wz){

u <- exp(wz[1])/(1+exp(wz[1]));

v <- exp(wz[2]);

return(c(u*v,(1-u)*v));

}

to.w <- function(pm) return(log(pm/(1-pm)));

to.pm <- function(w) return(exp(w)/(1+exp(w)));

ml.bpinf <- function(data,init=c(NA,NA)){

dev <- function(wz) -2*log(likbp.inf(data,to.ab(wz)));

if(is.na(init[1])) init <- log(c(1/max(data$dose),1));

res <- optim(init,dev);

return(list("ab"=to.ab(res$par),"mindev"=res$value));

}

ml.xpinf <- function(data,intxp=c(NA,NA)){

dev <- function(w) -2*log(likxp.inf(data,to.pm(w)));

if(is.na(intxp[1])) intxp <- log(c(1e-12/min(data$dose),10/max(data$dose)));

res <- optimize(dev,intxp);

return(list("pm"=to.pm(res$minimum),"mindev"=res$objective));

}

ml.inf <- function(data,intxp=c(NA,NA),init=c(NA,NA)){

crit <- qchisq(0.95,1); # chi square with 2 - 1 = 1 df

resxp <- ml.xpinf(data,intxp);

resbp <- ml.bpinf(data,init);

delta <- resxp$mindev - resbp$mindev;

if(delta < crit)

return(list("model"="exponential","df"=1,

"pm"=resxp$pm,"mindev"=resxp$mindev));

return(list("model"="beta-poisson","df"=2,

"ab"=resbp$ab,"mindev"=resbp$mindev));

}

ml.cond.ill <- function(data,init=c(NA,NA)){

dev <- function(wz) -2*log(lik.cond.ill(data,to.ab(wz)));

if(is.na(init[1])) init <- log(c(10/max(data$dose),1));

res <- optim(init,dev);

return(list("model"="hazard model","df"=2,

"ab"=to.ab(res$par),"mindev"=res$value));

}

lrtestpooling.inf <- function(data1,data2,data12,intxp=c(NA,NA),init=c(NA,NA)){

ml1 <- ml.inf(data1,intxp,init);

ml2 <- ml.inf(data2,intxp,init);

ml12 <- ml.inf(data12,intxp,init);

delta <- ml12$mindev - (ml1$mindev + ml2$mindev);

numdf <- ml1$df + ml2$df - ml12$df;

crit <- qchisq(0.95,numdf); # chi square with df = numdf

# cat(delta," ",numdf,"\n")

if(delta > crit) return("no");

return("yes");

}

lrtestpooling.inf.2 <- function(data1,data2,intxp=c(NA,NA),init=c(NA,NA)){

ml1 <- ml.inf(data1,intxp,init);

ml2 <- ml.inf(data2,intxp,init);

delta <- ml12$mindev - (ml1$mindev + ml2$mindev);

numdf <- ml1$df + ml2$df - ml12$df;

crit <- qchisq(0.95,numdf); # chi square with df = numdf

# cat(delta," ",numdf,"\n")

if(delta > crit) return("no");

return("yes");

}

lrtestpooling.ill <- function(data1,data2,data12,init=c(NA,NA)){

ml1 <- ml.cond.ill(data1,init);

ml2 <- ml.cond.ill(data2,init);

ml12 <- ml.cond.ill(data12,init);

delta <- ml12$mindev - (ml1$mindev + ml2$mindev);

numdf <- ml1$df + ml2$df - ml12$df;

crit <- qchisq(0.95,numdf); # chi square with df = numdf

# cat(delta," ",numdf,"\n")

if(delta > crit) return("no");

return("yes");

}

#

# Figure S1


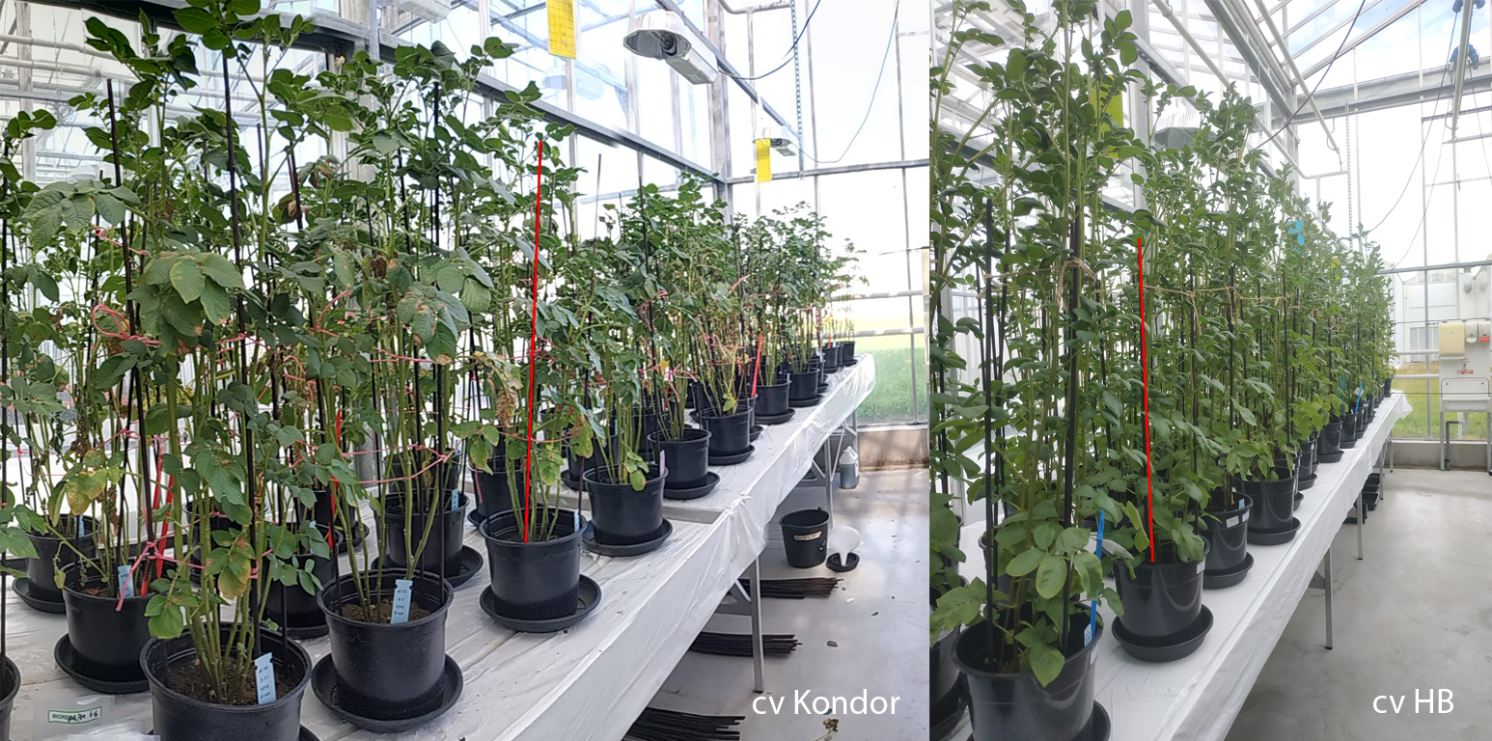


Figure S 1 Comparison of phenotypes of potato cultivars Kondor (left) and HB (right). Photo of cv Kondor was taken 74 days after planting and of cv HB 54 days after planting. One of the wooden sticks to support the plant is marked red in each graphic as the sticks had the same length and can be used as reference for length comparison. Cultivar HB grew longer than the wooden stick while cv Kondor has about the same length as the stick.

# Table S1

Table S 1 Weight of plant material collected from potato plants in greenhouse experiments used to re-isolate Ralstonia solanacearum.

|  | Samples of different plant material for re-isolation of *R. solanacearum*, weights in [g] | | | | | | | | | | | |
| --- | --- | --- | --- | --- | --- | --- | --- | --- | --- | --- | --- | --- |
|  | stem weight | | root weight | | number of tubers | | Weight of all progeny tubers per plant | | cut tuber weight (for re-isolation) | | soil weight | |
| **Dose** | **Kondor** | **HB** | **Kondor** | **HB** | **Kondor** | **HB** | **Kondor** | **HB** | **Kondor** | **HB** | **Kondor** | **HB** |
| Control | 12.5 | 8.8 | 10.0 | 6.0 | 11.0 | 2.2 | 183.2 | 48.1 | 2.9 | 0.8 | 2.8 | 5.7 |
| 5x10^2^ | 12.3 | 7.9 | 9.9 | 4.6 | 9.1 | 2.3 | 188.6 | 72.4 | 2.5 | 0.8 | 2.8 | 6.6 |
| 5x10^3^ | 12.9 | 8.5 | 9.9 | 5.2 | 11.0 | 2.3 | 190.7 | 66.7 | 2.9 | 0.8 | 2.8 | 6.9 |
| 5x10^4^ | 9.5 | 8.2 | 10.0 | 7.2 | 8.8 | 2.2 | 171.3 | 65.0 | 2.6 | 0.9 | 2.9 | 7.4 |
| 5x10^5^ | 14.1 | 8.0 | 11.4 | 9.0 | 9.8 | 2.3 | 182.5 | 63.0 | 2.6 | 0.9 | 2.8 | 6.3 |
| 5x10^6^ | 10.7 | 8.0 | 9.7 | 9.3 | 9.6 | 2.4 | 183.8 | 49.5 | 2.8 | 0.7 | 3.0 | 6.3 |
| 5x10^7^ | 11.9 | 9.7 | 8.8 | 7.4 | 9.5 | 2.6 | 180.4 | 56.3 | 2.8 | 0.9 | 3.1 | 6.7 |
| 5x10^8^ | 6.8 | 6.3 | 10.6 | 6.7 | 6.4 | 0.9 | 214.2 | 59.6 | 1.9 | 0.8 | 3.2 | 6.5 |
| 5x10^9^ | 8.1 | 5.4 |  | 5.5 | 3.3 | 1.0 | 43.8 | 34.2 | 0.5 | 1.3 | 3.6 | 6.4 |
| **total average** | **11.0** | **7.9** | **10.1** | **6.8** | **8.7** | **2.0** | **170.9** | **57.2** | **2.4** | **0.9** | **3.0** | **6.5** |

# References

Plummer, M. (2015). "rjags: Bayesian Graphical Models using MCMC. R package".).

R Core Team (2022). "R: A language and environment for statistical computing. R Foundation for Statistical Computing". (Vienna, Austria.).
